# Atomic-resolution imaging of surface and core melting in individual size-selected Au nanoclusters on carbon

**DOI:** 10.1038/s41467-019-10713-z

**Published:** 2019-06-13

**Authors:** D. M. Foster, Th. Pavloudis, J. Kioseoglou, R. E. Palmer

**Affiliations:** 1 Nanoscale Physics Research Laboratory, School of Physics and Astronomy, University of Birmingham, Birmingham, B15 2TT UK; 2 College of Engineering, Swansea University, Bay Campus, Fabian Way, Swansea, SA1 8EN UK; 3 Department of Physics, Aristotle University of Thessaloniki, University Campus, GR-54124 Thessaloniki, Greece

**Keywords:** Nanoparticles, Characterization and analytical techniques

## Abstract

Although the changes in melting behaviour on the nanoscale have long attracted the interest of researchers, the mechanism by which nanoparticles melt remains an open problem. We report the direct observation, at atomic resolution, of surface melting in individual size-selected Au clusters (2–5 nm diameter) supported on carbon films, using an in situ heating stage in the aberration corrected scanning transmission electron microscope. At elevated temperatures the Au nanoparticles are found to form a solid core-liquid shell structure. The cluster surface melting temperatures, show evidence of size-dependent melting point suppression. The cluster core melting temperatures are significantly greater than predicted by existing models of free clusters. To explore the effect of the interaction between the clusters and the carbon substrate, we employ a very large-scale ab initio simulation approach to investigate the influence of the support. Theoretical results for surface and core melting points are in good agreement with experiment.

## Abstract (web summary)

Much remains unresolved about the mechanisms of nanoparticle melting. Here, by observing and simulating individual gold nanoclusters at atomic level as they are heated inside an electron microscope, the authors find that the particle surface melts before the interior. Their experimental results in combination with the atomistic simulations confirm the coexistence of a liquid shell and solid core during melting, as well as size-dependent melting point suppression.

## Introduction

The behaviour of nanoparticles at elevated temperatures is interesting from a fundamental perspective (thermodynamics on the nanoscale) and is also relevant to functionalities such as catalysis. Specifically, it is now well-known that gold nanoparticles exhibit catalytic activity^1^. Understanding their morphology and atomic structure under relevant reaction conditions, such as high temperatures, may ultimately prove useful in the design of catalyst materials. The suppression of the melting point at the nanoscale^2^ is a longstanding issue. First predicted by Pawlow in 1908^3^ and detected experimentally by Takagi in 1954 via shape changes in the diffraction rings of a nanoparticle ensemble^4^, a $1/r$ dependence of melting temperature at the nanoscale seems to hold true for all except few materials^5,6^. Early experimental observations of melting point suppression in supported Au nanoparticles were reported in the seminal electron diffraction study by Buffat and Borel^7^, as well as in a transmission electron microscope (TEM) evaporation rate investigation by Sambles^8^. Subsequently, there have been several experimental studies of the high temperature behaviour of gold nanoparticles. However, at present there is only very limited single particle time-resolved data on melting, obtained by evaporation at fixed temperature^9^, with no data below 5 nm—the catalytic size regime^10^. In addition, because previously reported experimental studies of Au nanoparticle melting do not track individual particles in real space as the temperature is increased (instead they use static temperature evaporation or ensemble diffraction methods), the exact mechanism by which melting occurs remains unresolved, such as whether a surface liquid layer is formed.

There are several theoretical models for melting point suppression at the nanoscale. Pawlow’s theory is a thermodynamic model based on the triple point equilibrium of spherical solid and liquid particles of the same material and equal mass surrounded by their vapour. The liquid shell model, liquid nucleation and growth model, numerous molecular dynamics (MD) studies of Au^11–15^ and other metal^16–18^ nanoparticles predict the formation of a liquid shell as a mechanism for nanoparticle melting. The liquid shell model, first suggested by Reiss and Wilson^19^ and developed by others^20,21^, including Sambles^8^, is a thermodynamic model that assumes a solid core surrounded by a liquid shell of constant thickness in the proximity of the melting temperature. The liquid nucleation and growth model, proposed by Couchman and Jesser^22^, is based on nucleation theory and describes melting as the nucleation of a liquid shell on the surface of the nanoparticle which then spreads into the core until a critical radius is reached and the whole particle melts. Thus, the model predicts a melting region in which there is solid–liquid coexistence. There has been no experimental observation of the existence of a liquid shell prior to melting for Au nanoparticles. Young et al.^23^ reported surface roughening (amorphous regions) in 10.2 nm diameter Au particles at 600 °C, however, a liquid shell was not observed. Such a solid core, liquid shell structure and thus solid–liquid coexistence have been reported in electron microscopy investigations of embedded lead^24^, and polymer-capped platinum particles^25^, but without atomic resolution, and most recently for large gallium nanoparticles at fixed (room) temperature^26^.

Here we observe the behaviour of individual, supported, size-selected Au nanoclusters (≤5 nm) in real space with atomic resolution as their temperature is increased from 20 °C up to 1000 °C. We employ an in situ heating stage in the aberration-corrected scanning transmission electron microscope (ac-STEM). Single particle measurements of the surface melting temperature on amorphous carbon supports are made by a unique method in which shape changes of the nanoparticles are observed as the temperature is ramped. The results show an approximately $1/r$ dependence on the surface melting temperature, in agreement with Pawlow’s triple point model and the liquid nucleation and growth model. However, the cluster core-melting temperature is much higher than predicted by the models of melting point suppression. The ac-STEM images further reveal the formation of a quasi-liquid shell that persists over a range of temperatures. Large-scale ab initio MD simulations of cuboctahedral 561-atom nanoclusters at temperatures in the experimental range address the previously neglected effect of the support on the melting of the nanoparticles. The theoretical results support the experimental observations of solid core-liquid shell coexistence and agree with the measured surface and core-melting points if the surface is understood to constrain the facet bound to it.

## Results

### Experimental

**Fig. 1:**
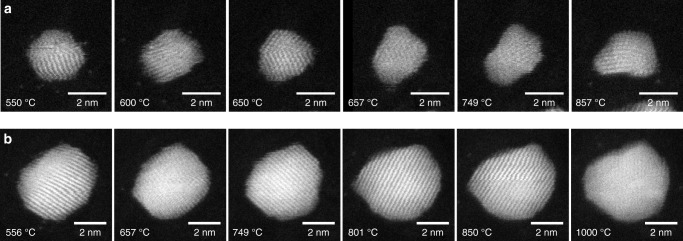
Shape changes in Au nanoclusters at high temperatures. HAADF STEM images of **a** an individual Au_561_ particle at high temperature (550–857 °C) and **b** an individual Au_2530_ particle (556–1000 °C)

Figure 1a shows a Au_561_ particle that is heated incrementally from 550 to 857 °C. The frames shown in the figure are taken from a series of 22 high-angle annular dark-field (HAADF) STEM images of this particle. The shape of the particle is first changed at 657 °C, where there is a protrusion from the cluster surface, which we take to be indicative of surface melting. For this particle the surface melting temperature is recorded as 654 ± 4 °C. Similarly, Fig. 1b shows a Au_2530_ particle formed by aggregation heated from 556 to 1000 °C. In this case the change in shape first appears at 801 °C, and the surface melting temperature is recorded as 800 ± 1 °C (i.e. between images recorded at 799 and 801 °C). The errors stated here do not include the potential systematic error of <5% arising from the heating chip calibration.

There are at least two ways in which the melting temperature of a nanoparticle could be measured using in situ heating in the STEM. The first, as employed conventionally, would be to use the loss of atomic structure in the images as an indication of melting^9^. The second, proposed here, is to use changes in shape—a method that arises directly from the quality of data collected here (although shape changes have been previously used to describe melting in field emission microscopy experiments^27^). In our study the criterion of loss of atomic structure is not suitable due to the time resolution of the experiments (each image taking 5.4 s to record). The phenomenon of quasi-melting below the melting point, where the particle structure rapidly fluctuates, has been reported in both experimental and theoretical studies^28,29^. If this were to occur, the rapidly fluctuating particle would likely appear amorphous in the recorded STEM image and could be misinterpreted as melted. The shape change method attributes changes in shape in the projected images to diffusion of atoms in the molten surface layers. MD simulations of cluster surface melting show that diffusion of atoms results in the formation of anisotropic cluster shapes^11,13,15^, such as those seen in our experimental STEM images. The association between shape changes and peripheral melting in these papers is notable, and consistent with our interpretation of our observations. At still more elevated temperatures more dramatic shape changes are sometimes found.

**Fig. 2:**
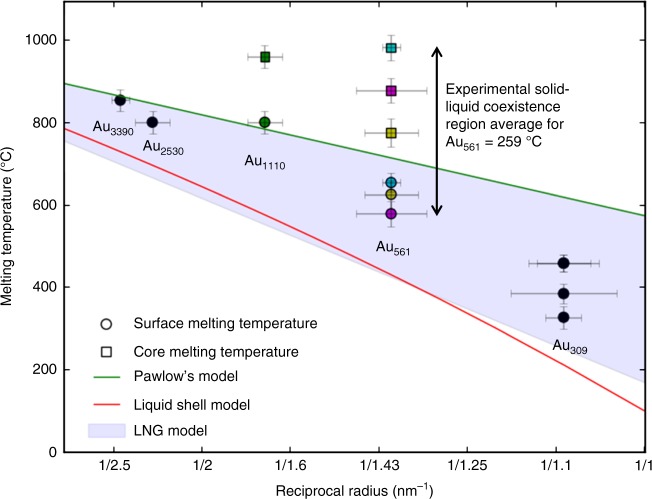
Experimental, single particle measurements of melting point suppression in Au nanoparticles. Scatter points represent the experimental data: circles show surface melting temperatures and squares show core-melting temperatures. The cluster sizes (number of atoms) are indicated on the plot and the corresponding core and surface melting temperatures have the same colour. The solid green line is Pawlow’s model from ref. ^49^, the solid red line is the liquid shell model from ref. ^8^ and the blue region is the liquid nucleation and growth model melting sector from refs. ^22, 50^. The error bars on the melting temperatures are systematic errors arising from the temperature window, the temperature stability of the MEMS heating chip and the 5% heating chip calibration error

Figure 2 shows the results of our single particle analysis of the surface melting temperature of the Au clusters as a function of the reciprocal radius. A clear inverse correlation is seen. Where possible, cluster core (i.e., complete) melting temperatures are also shown, measured by observing the loss of core atomic structure. The scatter points are measurements of size-selected Au_309_ and Au_561_ particles and aggregated Au_1110_, Au_2530_, and Au_3390_ particles. The error on the surface melting temperature includes both the error arising from the temperature window and the 5% heating chip calibration error, as described in the Methods section. The particle radius was calculated by making four separate measurements of the average particle diameter, then taking the mean value; the error is given by the maximum and minimum of these values, shown by error bars in the figure.

Also shown in Fig. 2 are Pawlow’s triple point model^3^, the liquid shell model^8^ and the liquid nucleation and growth (LNG) model^22^ are also plotted for comparison (for details see Supplementary Methods and Supplementary Fig. 1). For the liquid shell model, the smallest possible shell thickness of 2.7 Å (the atomic diameter) is used. Pawlow’s model is for complete (i.e. including core) particle melting; thus, it is reasonable to assume that our surface melting measurements should fall below those predicted by this model. This is true for all but one particle (Au_1110_). However, the experimental core-melting temperatures are much higher than predicted. The liquid shell model predicts significantly lower whole particle melting temperatures than Pawlow across our size range, even more so for small particles. The melting temperatures predicted are much lower than the observed core and even the observed surface melting temperatures. Moreover, additional ac-STEM images (see below) show a liquid shell thickness which varies between particles and is generally greater than the value of 1 atomic layer embedded in the theory curve. Increasing the shell thickness in the liquid shell model amplifies the melting point suppression for small particles sizes, which is not mirrored by the experimental data. Indeed, the data are well fitted by a straight line ($T_m = - 904/r + 1518$, *R*^2^ = 0.9). The liquid nucleation and growth model predicts a melting region; the lower boundary is the onset of surface melting and the upper boundary is the point by which the complete particle melts. Within this region solid and liquid phases coexist. Again, our surface and core-melting temperature measurements are higher than those predicted, but this LNG model is in the best qualitative agreement with our data. It captures the experimental result that surface melting precedes core melting, so there is a solid–liquid coexistence which occurs over a range of temperatures. The size of our solid–liquid coexistence region is in very good agreement with the coexistence region predicted by the LNG model, deviating by ~20 °C. The different melting temperatures observed for the different 561-atom nanoclusters is a noteworthy result of our paper. The differences between the core-melting temperatures in Fig. 2 could be due to specific structural characteristics of each examined nanoparticle, whether intrinsic (e.g. we sometimes observe twin structures as shown by the power spectrum fast Fourier transform (FFT) image in Supplementary Fig. 2, and also observable by eye) or extrinsic (such as different orientations of the cluster on the support).

To check the surface melting temperatures obtained from the observed onset of the cluster shape changes, we developed a numerical approach, based on tracking the curvature along the perimeter of the nanoclusters. This is described in detail in the Supplementary Methods and shown in Supplementary Fig. 3 for a 561- and a 1110-atom nanocluster. This approach is in good agreement with the observations by eye, yielding results which are either identical or deviate by only a small margin. The results of the two methods for the 561-, 1110-, 2530- and 3390-atom nanoclusters are shown in Table 1. The average of the melting temperature for the 561-atom nanoclusters obtained by observation is 619 °C, while the average by the numerical method is 603 °C, a difference of only 16 °C.

**Table 1:** A comparison between the surface melting temperatures obtained by the two methods

NP size (atoms)	Surface melting temperature by eye (°C)	Surface melting temperature by numerical method (°C)
561	578	578
561	625	653.5
561	653.5	578
1110	800	800
2530	800	825.5
3390	854	825.5

**Fig. 3:**
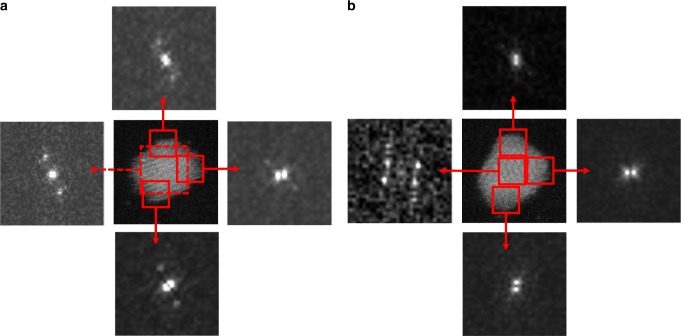
Power spectrum FFT images of various regions of a Au_561_ nanocluster. The nanocluster at **a** 600 °C and **b** 657 °C

Furthermore, the coexistence of an amorphous melted surface with a solid crystalline core can be verified by power spectrum FFT images of multiple areas of each nanoparticle. In Fig. 3, experimental images of a nanoparticle at 600 and 657 °C are shown with the power spectra obtained from the marked regions. The additional spots in Fig. 3a power spectra indicate a degree of crystallinity in the shell. In Fig. 3b, it is obvious that the central part of the nanoparticle is still crystalline, while the surface areas are not (lack of peripheral spots).

**Fig. 4:**
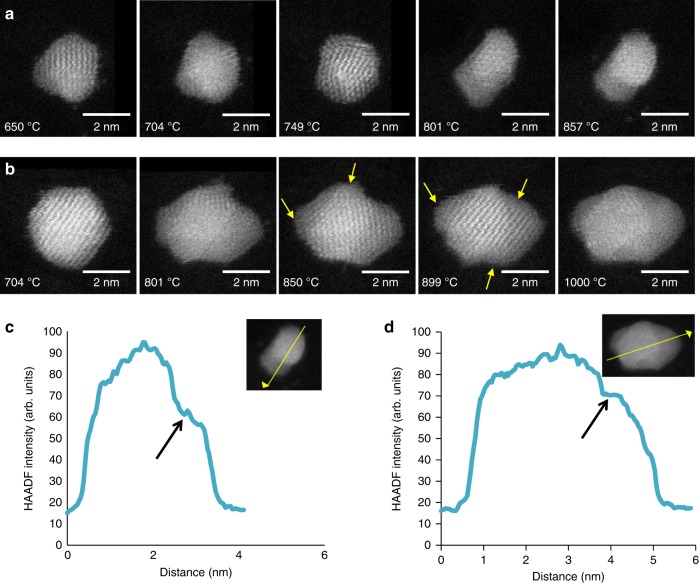
The formation of a liquid shell in Au nanoclusters at high temperatures. **a** HAADF STEM images of an Au_561_ particle at high temperatures (650–857 °C). **b** HAADF STEM images of an Au_1110_ particle at high temperatures (704–1000 °C). Amorphous regions at the edges of the particle are highlighted by yellow arrows. **c** A line profile plot of the HAADF intensity across the particle in (**a**) at 857 °C and **d** a line profile plot of the HAADF intensity across the particle in (**b**) at 1000 °C. Black arrows on the HAADF intensity plots illustrate amorphous regions. Yellow arrows on the inset HAADF images indicate the direction and location of the line profile

Figure 4 shows two series of HAADF STEM images, which further demonstrate the formation of a quasi-liquid non-uniform layer at the cluster surface and the coexistence of solid and liquid phases at high temperatures. Figure 4a, for a Au_561_ particle above its surface melting point, exhibits a surface protrusion at 650 °C indicating the surface is molten. At 800 °C, a complete liquid shell-solid core structure is visible. Figure 4b shows a cuboctahedral Au_1110_ particle which is solid at 704 °C and exhibits dramatic shape change at 801 °C. Again, we see a solid core-uneven liquid shell structure. The corresponding profile plots in Fig. 4c, d indicate the fact in atom density at the edge of the solid core. In both clusters the core atomic structure persists after surface melting. The larger Au_1110_ particle shows some face-centred cubic (fcc) structure in the shell too. We found no evidence of a rapidly fluctuating quasi-molten state^23,28–30^ nor an icosahedral or icosahedral solid–liquid coexistence state prior to melting^12,31,32^.

The degree of formation of the solid core-liquid shell structure (as seen in Fig. 4) does vary from cluster to cluster, but in all cases melting initiates at the surface. The notable delay between cluster surface and cluster core melting is consistent with the liquid nucleation and growth model, however, a comparison of our experimentally measured critical core radii (Supplementary Fig. 1) with the model show some significant differences. Experimentally, we obtain a notably lower limit on the critical core radius and a much higher upper limit on the core-melting temperature (see Fig. 2).

### Theoretical

**Fig. 5:**
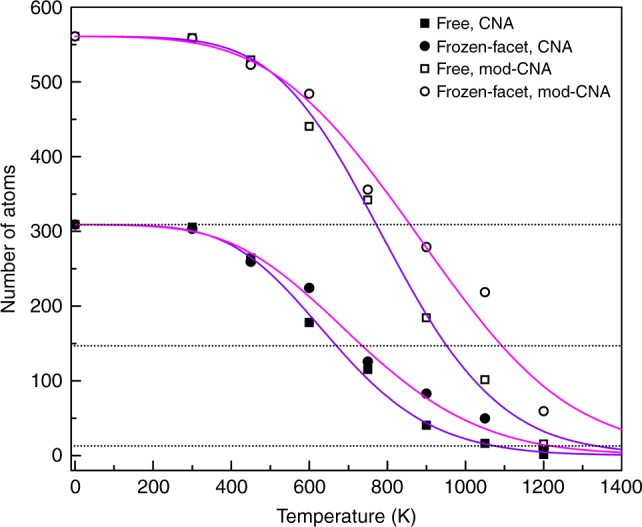
Theoretical melting point suppression in cuboctahedral Au_561_ nanoclusters. Scatter points represent statistical averages for the number of crystalline atoms obtained by the CNA (solid points) and modified CNA algorithms (hollow points) as a function of temperature for two families of simulations: nanoclusters all of whose atoms are free to move (squares), and nanoclusters having atoms of one (100) facet fixed in place during the simulations (circles). The curves are sigmoidal fits to the data. Horizontal dotted lines denote the 309-, 147 and 13-atom limits

An intriguing issue raised by the mismatch between the experimental and conventional models of nanoscale melting is the influence of the substrate. In order to explore the effect of the carbon support on the melting of the clusters, we adopt an ab initio computational approach with two sets of density functional theory molecular dynamics (DFT-MD) simulations: one where all the atoms of the nanocluster are free to move and one where the atoms of one (100) facet of the nanocluster are fixed in place during the simulations. We proceed to extract the degree of crystallinity of the nanoclusters during the last steps of the simulations. The results for all the investigated cases and analysis algorithms applied are shown in Fig. 5. The calculations give a sizable difference in the behaviour and melting temperatures for the free nanocluster and the nanocluster with a frozen facet. The two types of nanoclusters are almost identical at room temperature but display major differences at elevated temperatures. The freezing of the facet, i.e. the simulated influence of the support, is found to delay the melting of the nanocluster considerably.

We use the criterion that surface melting occurs when the count of the atoms recognised as fcc and their 12 nearest neighbours is <309, i.e. one atomic layer is melted. This critical point is identified at a temperature of 500 °C for the free and 586 °C for the frozen-facet Au_561_ clusters, with the average of the experimental measurements at 619 °C lying closer to the frozen-facet case. The surface melting temperature difference between the free and frozen-facet nanoclusters is 86 °C. If we proceed further and check where the aforementioned count is lower than 147 atoms, we get temperatures of 680 and 822 °C for the free and frozen-facet clusters, respectively. For cluster core melting we use the criterion that the number of atoms that belong to an fcc arrangement is lower than 13, which corresponds to the smallest possible crystalline magic-number nanocluster. Using this threshold, we observe cluster core melting at 807 and 951 °C for the free and frozen-facet nanoclusters, respectively, a temperature difference of 144 °C. These results are in good agreement with the average core-melting temperature for 561-atom nanoclusters shown in Fig. 2, which is 878 °C, between the core-melting temperatures of the free and frozen-facet nanoclusters.

**Fig. 6:**
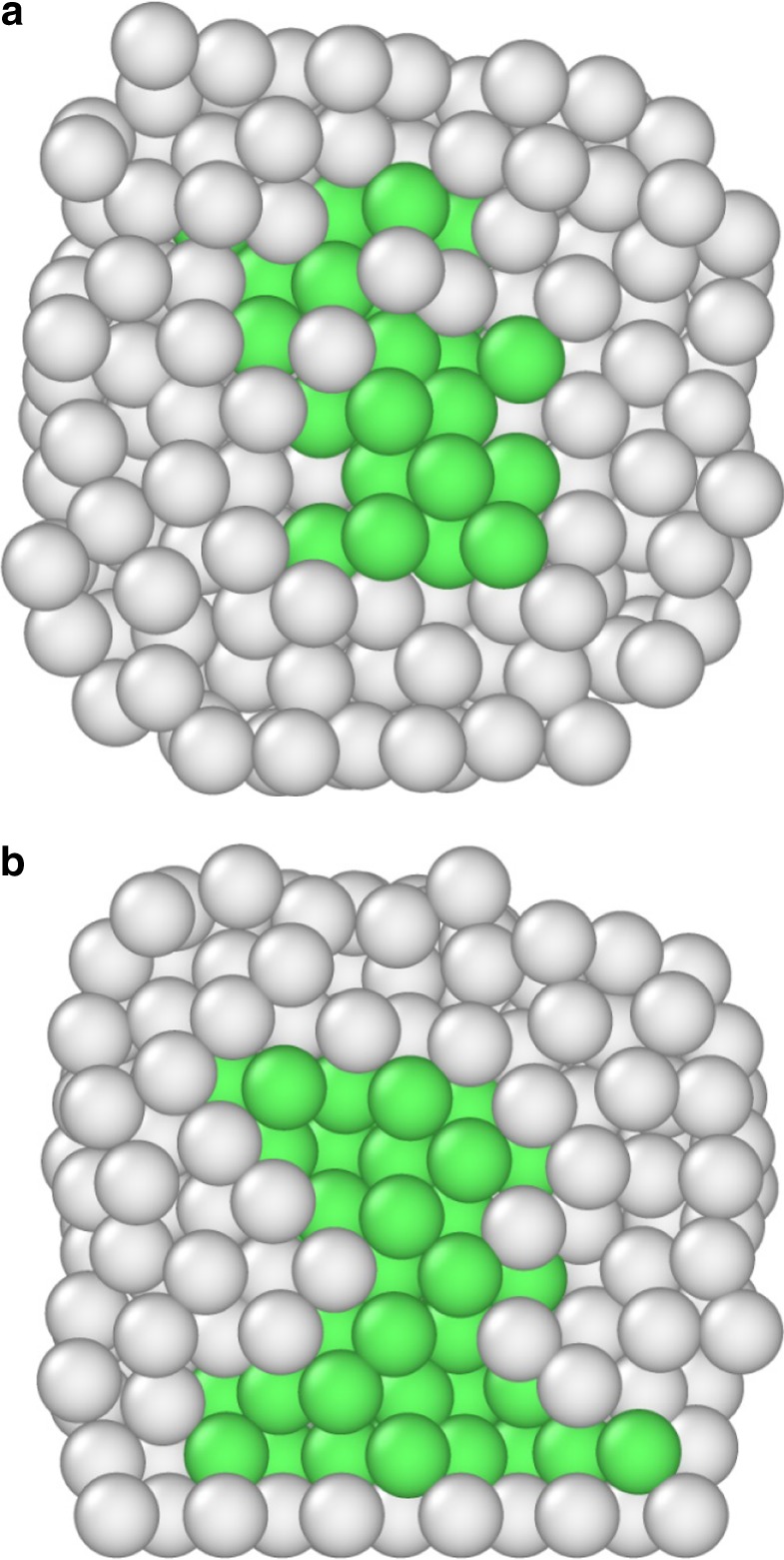
Cross-sections of Au_561_ nanoclusters at elevated temperatures. **a** A free and **b** a frozen-facet nanocluster at 627 °C. Green spheres denote atoms that are recognized as fcc, while grey spheres are atoms found to be non-crystalline. The bottom layer in (**b**) is the frozen layer. At this temperature the core is approximately double in size in (**b**) compared with (**a**) (85 atoms and 43 atoms, respectively) and displaced towards the frozen facet

We also note that a solid core is observed in the nanoclusters after the melting process, located at the centre of the nanocluster for the free nanoclusters, as shown in Fig. 6a. The core is of larger size and displaced towards the frozen facet in the other nanoclusters, as shown in Fig. 6b, revealing another effect of the support on the melting process. The solid–liquid coexistence interval is found to be 307 and 365 °C for the free and frozen-facet cases, respectively, higher than the 259 °C found by the experimental observations shown in Fig. 2. This is possibly due to the lower surface melting temperatures obtained by the simulations compared with the experiments.

## Discussion

We have employed in situ heating experiments on sub-5 nm size-selected Au nanoparticles supported on a-C in the aberration-corrected STEM, demonstrating melting initiated at the cluster surface as well as size-dependent melting point suppression. The surface melting temperature of the nanoclusters was measured using a method—the observation of nanoparticle shape changes at high temperatures. We have observed single particle surface melting in real space, identified the formation of a solid core-liquid shell structure at elevated temperatures and proved the coexistence of solid and liquid phases. The mechanism is broadly consistent with the liquid nucleation and growth model, with good agreement for the solid–liquid coexistence interval, but notably higher surface and cluster core-melting temperatures.

The influence of the support is apparent from large-scale ab initio simulations that mimic the effect of cluster adsorption by a frozen-facet approach. Quantitative agreement between experiment and theory is obtained when the surface is introduced in this way. The theoretical results confirm and illuminate our interpretation of the experimental results. Specifically, the melting point suppression and the coexistence of a melted shell and a crystalline core at elevated temperatures are both confirmed. The introduction of the frozen-facet approximation to cluster adsorption leads to core-melting temperatures for both the free and frozen-facet clusters which are notably higher than the standard models but in good agreement with the experimental results. Moreover, we find that the solid core in the coexistence region is enlarged due to the effect of the support, and displaced towards the surface. We therefore conclude that the main origin of the deviations of the experimental work from the previous models lies in the effect of the cluster-support interaction and is well captured by the frozen-facet model. Since the Au particles studied here lie in the catalytically active size regime, the observations also raise the question of whether the liquid surface affects the catalytic activity at high temperatures.

## Methods

### Experimental

The size-selected Au nanoclusters were prepared using a magnetron sputtering, gas aggregation cluster source^33^ with lateral time of flight mass filter (*M*/∆*M* = 22)^34^. Clusters containing either 309 ± 7 or 561 ± 13 Au atoms were deposited onto amorphous carbon membranes of the heating chips, using low deposition energy (soft landing^35^) to preserve their original structures. STEM imaging was performed using a 200 keV JEOL 2100F instrument with spherical aberration corrector (CEOS). A HAADF detector, with inner collection angle of 62 mrad, as well as a bright field detector were employed for imaging. Use of the HAADF detector enabled any aggregate particles to be sized accurately by using the size-selected clusters as ‘mass-standards’^36^. Imaging was performed at an electron dose of $2.5 \times 10^4\,e^ - \cdot {\mathrm{{\AA}}}^{ - 2} \cdot {\mathrm{frame}}^{ - 1}$ for the 309-atom nanoclusters and $4.8 \times 10^4\,e^ - \cdot {\mathrm{{\AA}}}^{ - 2} \cdot {\mathrm{frame}}^{ - 1}$ for the 561-, 1110-, 2530- and 3390-atom nanoclusters.

For in situ heating a high temperature heating holder (DENS Solutions Wildfire) was used in conjunction with MEMS-based heating chips. The chips featured 5-nm-thick amorphous carbon membranes, on which the Au nanoparticles were deposited. They were heated by applying a current to a metal heater coil embedded in the chip. Heating experiments were performed by STEM imaging of individual particles at incrementally increasing temperatures. The surface melting temperature of the nanoparticles was determined by observing the onset of shape changes (shape changes due to rotations of the particle were excluded by comparison against simulation atlases^37^ showing particles at different angles of orientation). When a shape change was observed, the surface melting temperature for that particle was recorded as the average between the temperatures of the last observed particle of the original-shape and that of the first changed shape. In order to check the assignments of the surface melting temperatures obtained from visual inspection of the images, a numerical method was developed to assess protrusions and dents on the nanoclusters. It tracks the curvature along the perimeter of the nanoclusters. The method identifies surface alterations which show significant deviation from quasi-spherical shape. The occurrence of such deviations is associated with surface melting of the nanoclusters (further details on the method are given in the Supplementary Methods).

The precision of the measured surface melting temperature is a function of the temperature window in which the shape change occurs and the temperature stability of the MEMS heating chip, which is essentially negligible in comparison (<0.1 °C). Another source of error arises from the calibration of the heating chip; there may be a systematic error—an offset of up to 5% on the stated temperature. This systematic error effects the accuracy of all temperature measurements. However, so long as measurements are made using the same heating chip the general trend (melting temperature vs particle size) should not be affected.

### Theoretical

The DFT-MD calculations were performed using the Vienna Ab initio Simulation Package (VASP)^38,39^ under the local density approximation (LDA)^40^ with projector-augmented wave (PAW) pseudopotentials^41,42^. A canonical ensemble under Born–Oppenheimer MD was simulated with a time step of 2 fs to integrate the equations of motion using the algorithm of Nosé^43–45^, with a Nosé mass equal to 40 time steps. The energy cut-off of the plane-wave basis set was 240 eV and the tolerance for self-consistency for the electronic steps was set at 10^−6^ eV. The lattice constant of the 8-atom Au unit cell was found to be equal to 4.057 Å. The Brillouin zone was sampled using a Γ-centred 5 × 5 × 5 mesh for the unit cell, scaled accordingly for the large-scale calculations.

Magic-number cuboctahedral nanoclusters, chosen on the basis that they have been proven to be of the greatest abundance and lower energy amongst the three magic-number structural isomers of nanoparticles^46^, that consisted of 561 atoms were constructed for the simulations. After an initial structural relaxation to obtain the ground-state configuration for the nanoclusters at *T* = 0 K, separate NVT simulations of the temporal behaviour were performed at temperatures from 300 to 1200 K at 150 K intervals. The total duration of each simulation was 2.4 ps. The optimized width of the vacuum surrounding the nanoclusters was equal to 14 Å in all directions, which ensured that there was no interaction between the nanocluster and its nearest image and took into account the thermal expansion identified during the simulations.

The simulation approach we followed in this work was based on two sets of DFT-MD simulations. In one set all the atoms of the nanocluster were free to move (free clusters). In the other the atoms of one (100) facet of the nanocluster, consisting of 36 atoms for the 561-atom nanocluster, were fixed in place during the simulations to capture the effect of the surface on the melting of the clusters. A Common Neighbour Analysis (CNA)^47^ using a fixed cut-off radius equal to 3.4629 Å was performed with the OVITO software^48^ in order to extract the degree of crystallinity of the nanocluster as a qualitative measure of the melting process. Statistical averages were collected over the last 600 steps of the simulations. It should be noted that the atoms belonging to the outer layer of the nanocluster, i.e. 252 atoms for the 561-atom nanocluster, were always recognized as non-crystalline by the CNA. In order to assess the surface melting in a better way, the atom selection was expanded to include the atoms recognised to be in an fcc crystalline arrangement by the CNA algorithm and each of their 12 nearest neighbours (each atom was counted only once). In this way the initial count was equal to 561. Thus, the modified CNA approach was used to extract the surface melting temperatures, whereas the CNA approach was used to extract the cluster core-melting temperatures. We adopt the criterion that surface melting occurs when the count of the atoms obtained by the modified CNA approach is <309, i.e. one atomic layer is melted, while for cluster core melting we adopt the criterion that the count of atoms obtained by the CNA approach is lower than 13.

### Reporting summary

Further information on research design is available in the Nature Research Reporting Summary linked to this article.

## Supplementary information

Supplementary Information

Reporting Summary

## Data Availability

The datasets generated during and/or analysed during the current study are available in the Zenodo repository, 10.5281/zenodo.2649945. Supplementary Information is available in the online version of the paper.
