# Supplementary material for: Atomic-resolution imaging of surface and core melting in individual size-selected Au nanoclusters on carbon
Source: Nat Commun. 2019 Jun 13;10:2583. doi: 10.1038/s41467-019-10713-z (PMC6565695; doi:10.1038/s41467-019-10713-z)
Supplement: Supplementary file 1 — Supplementary Information [file 41467_2019_10713_MOESM1_ESM.pdf]

Supplementary Information for:

Atomic-resolution imaging of surface and core melting in individual size-selected Au nanoclusters on carbon

Foster et al.

Supplementary Methods

Melting Models

Below are the equations and constants used for plotting melting models in Fig. 2 and Supplementary Figure 1.

Pawlow's Triple Point Model

$$T_m = T_0\left(1 - \frac{2V_s}{Lr}\left(\sigma_s - \sigma_l\left(\frac{\rho_s}{\rho_l}\right)^{\frac{2}{3}}\right)\right) \tag{1}$$

Equation from Supplementary Reference (1)

Liquid shell model

$$T_m = T_0\left(1 - \frac{2V_s}{L}\left(\frac{\sigma_{sl}}{r-t} - \frac{\sigma_l}{r}\left(1 - \frac{\rho_s}{\rho_l}\right)\right)\right) \tag{2}$$

Equation from Supplementary Reference (1)

LNG model lower limit

$$T_m = T_0\left(1 - \frac{3\sigma_{sl}V_s}{Lr}\right) \tag{3}$$

Equation from Supplementary Reference (2)
The upper limit is given by Pawlow's model, Supplementary References (1) and (3).

LNG model critical radius

$$r_c = \frac{2\sigma_{sl}V_sT_0}{L(T_0 - T)} \tag{4}$$

Equation from Supplementary Reference (3)

In the above:

T_m = melting temperature
T_0 = bulk melting temperature
V_s = molar volume of the solid
L = molar latent heat
r = particle radius
σ_s = surface tension of the solid
σ_l = surface tension of the liquid
ρ_s = mass density of the solid
ρ_l = mass density of the liquid

$\sigma_{sl} = \sigma_s - \sigma_l$
t = liquid shell thickness
r_c = critical radius

The values used for plotting the melting models are noted in Supplementary Table 1.

Numerical methodology

Using the Image-Pro Plus image analysis software, we track the perimeter of the nanoclusters in the using an intensity range selection from 45 to 255 for all images. Consequently, we find the intensity weighted centre of mass of the nanoclusters (centroid) and place a concentric linear grid mask with 36 segments on the centroid. We measure the coordinates of the points where the grid mask lines cross the tracked perimeter and proceed to calculate for each pair of points the curvature k_{norm} according to:

$$k_{norm} = \frac{dR_{12}}{d\theta_{12}} * \frac{180}{\pi\left(\frac{r_1 + r_2}{2}\right)} \tag{5}$$

where dR_{12} is the distance between points P_1 and P_2, $d\theta_{12}$ the internal angle of said points and the centre of mass and r_1 and r_2 their respective distances from the center of mass.
We use a value of 1.30 as an indication of a big enough shape change (protrusion or dent) in the nanocluster profile to signify surface melting, with the exception of the largest nanocluster of 3390 atoms where the biggest value registered was ~1.28. We follow the same procedure as in the experimental observations, i.e. when such a shape change is recorded, we register the surface melting temperature as the average between the frame where the change occurred and the last frame of the original shape.

Supplementary Figures

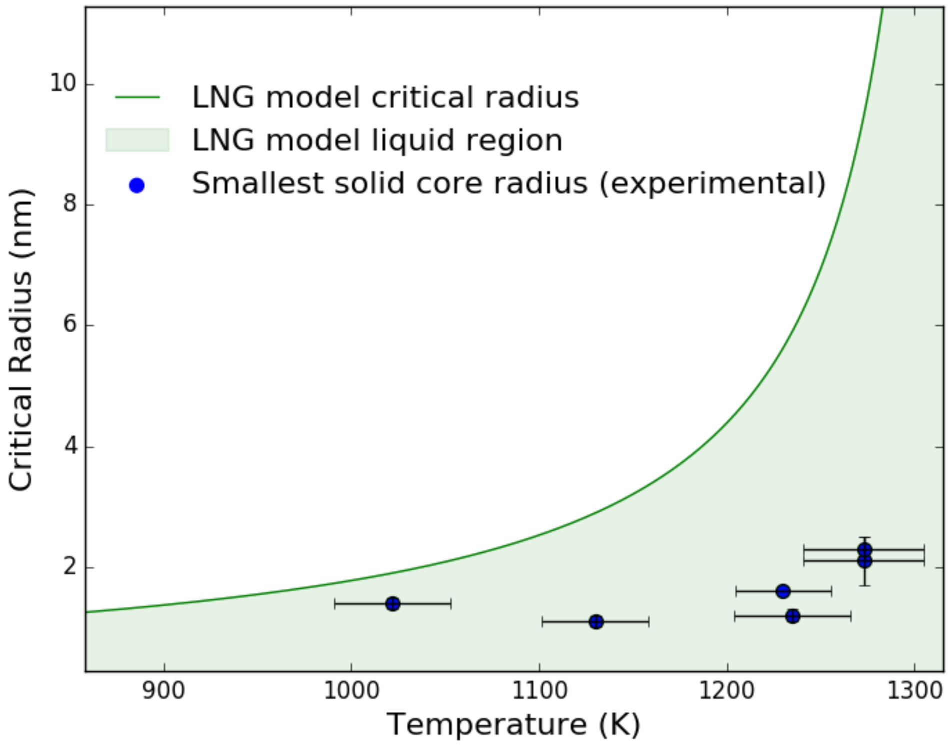

Supplementary Figure 1. The liquid nucleation and growth model critical radius compared with experimentally measured solid core radii. The LNG model critical radius is plotted (green line), the region below the curve (filled green) is the liquid region (core and surface) as predicted by the model. Experimental measurements of the smallest solid core sizes observed are also plotted (blue scatter points). The error bars on the temperatures are systematic errors arising from the temperature window, the temperature stability of the MEMS heating chip and the 5 % heating chip calibration error.

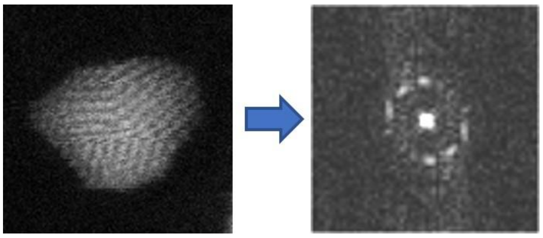

Supplementary Figure 2. Power spectrum FFT image of a 561-atom nanocluster at 704 °C. Additional spots surrounding the central spot in the right image are characteristic of a twin structure, also observable by eye in the left image.

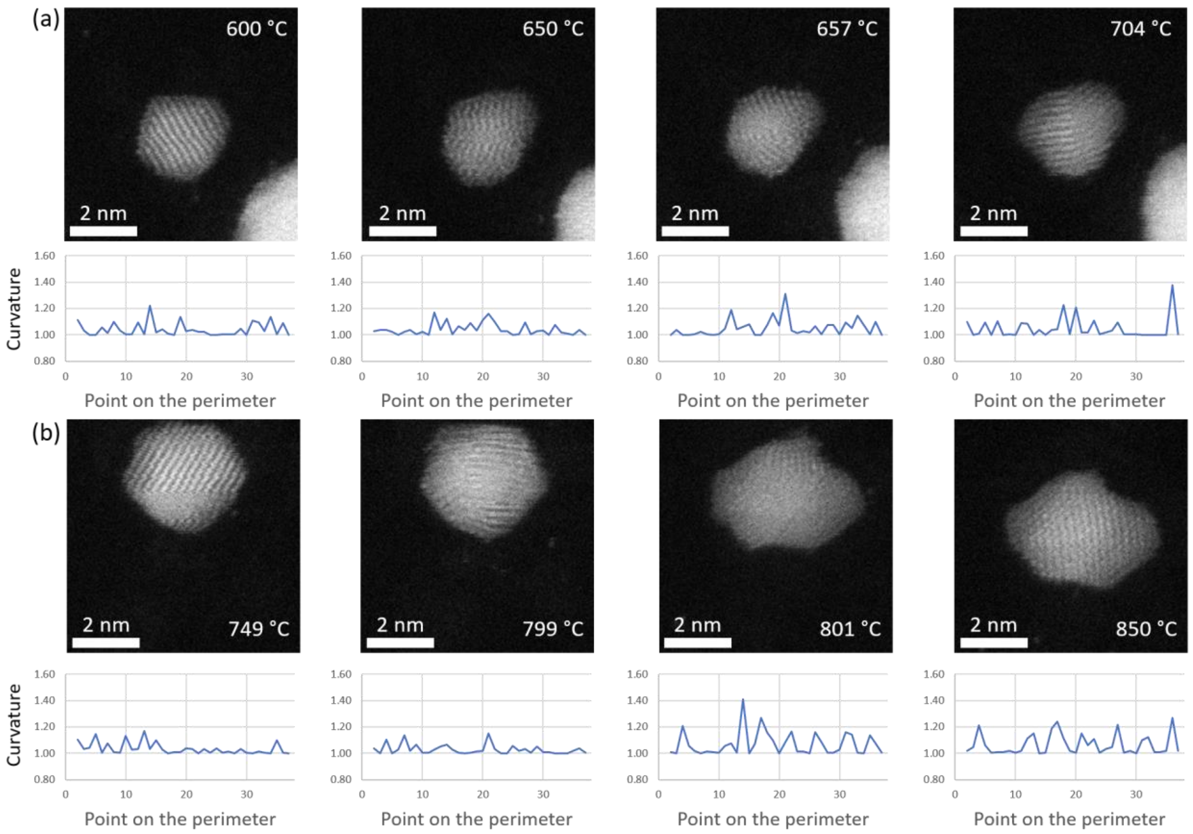

Supplementary Figure 3: The curvatures along the perimeter of a (a) 561- and (b) 1110-atom nanocluster. The horizontal axes on the plots are points along the perimeter, while the vertical axes are the curvatures. Surface melting according to the numerical method is observed at the 657 °C frame for the 561- and the 801 °C frame for the 1110-atom nanoclusters respectively.

Supplementary Tables

Symbol	Value	Reference
T_0	1336 K	1
V_s	10.7109×10^{-6} m^3/mol	3
L	12362 J/mol	1
σ_s	1.4 J/m^2	1
σ_l	1.13 J/m^2	1
ρ_s	18400 kg/m^3	1
ρ_l	17280 kg/m^3	1

Supplementary Table 1. Constants used for plotting the melting models in Supplementary Figure 1.

Supplementary References

1. Chushak Y. G. & Bartell L. S. Melting and freezing of gold nanoclusters. *J. Phys. Chem. B* **105**, 11605–11614 (2001).
2. Couchman P. R. & Jesser W. A. Thermodynamic theory of size dependence of melting temperature in metals. *Nature* **269**, 481–483 (1977).

3. Guenther G. & Guillon O. Models of size-dependent nanoparticle melting tested on gold. *J. Mater. Sci.* **49**, 7915–7932 (2014).
